# Development and psychometric properties of clinical learning environment scale for Chinese nursing students

**DOI:** 10.1186/s12909-024-05087-w

**Published:** 2024-01-31

**Authors:** Yun Xu, Yi Zheng, Huimei Wang, Fang Huang

**Affiliations:** 1 School of Nursing, Nanjing University of Chinese Medicine, Nanjing, China; 2 Institute of Education, Nanjing University, Nanjing, China

**Keywords:** China, Nursing students, Clinical learning environment, Scale development, Scale test

## Abstract

**Objective:**

To develop a clinical learning environment scale for Chinese nursing students and test its reliability and validity.

**Methods:**

Based on Moos social environment theory, qualitative interviews and expert consultations were used to develop a pretest version of the Chinese Nursing Students’ Clinical Learning Environment Scale. With a convenience sampling method, 255 and 1582 Chinese nursing students were selected as the prediction and validation samples, respectively, from December 2022 to March 2023. Exploratory and confirmatory factor analyses were conducted to determine the construct validity.

**Results:**

The scale consisted of 19 items. Exploratory factor analysis revealed three sub-scales, named goal orientation, interpersonal relation, and learning support, which explained 71.500% of the total variance. The results of the confirmatory factor analysis showed that the GFI was 0.848, the AGFI was 0.806, the RMSEA was 0.090, the RMR was 0.041, the NFI was 0.910, the IFI was 0.916, the CFI was 0.916, the PCFI was 0.798, and the PNFI was 0.793. The reliability values of the three dimensions were 0.870, 0.858, and 0.943, respectively, and the convergent validity values were 0.574, 0.603, and 0.625, respectively.

**Conclusion:**

The reliability and validity of the dimensions of the Chinese Nursing Students’ Clinical Learning Environment Scale are acceptable, and the scale can be used as a useful tool for measuring the clinical learning environment of Chinese nursing students.

## Introduction

The clinical learning environment involves the integration of all types of factors that affect the learning effectiveness of students during the learning process, including hospital culture, teaching staff, doctor‒patient relationships, teaching resources, learning opportunities, and other clinical staff. An interactive network is composed of all influencing factors that are interrelated and interactive and is an integral part of the learning experience [1]. The clinical learning environment provides students with opportunities to apply and practice theoretical knowledge in a real environment, realize their professional socialization, build professional confidence, and promote their career role transformation [2, 3]. Compared to the general learning environment, the clinical learning environment is more closely integrated with the social environment, which makes it more specific and complex and even more difficult for teachers to predict and manage. Moreover, the clinical learning environment is a working environment in which patients serve as clear service objects and students are not the core subject of teaching; this environment is significantly different from the traditional school learning environment.

In the 1980s, scholars paid more attention to the clinical learning environment of nursing students and analyzed the environment’s existence, specificity, and importance. However, the understanding of the environment was limited by the interactions between clinical teachers and students, and full attention has not been given to the comprehensive factors affecting students [4]. Since the 1990s, researchers have further discussed the connotation of the clinical learning environment for nursing students, and they have proposed components such as organizational support, a personalized learning environment, access to educational resources, the team atmosphere, participation in nursing work, social support, supervision relationships, management quality, and ward culture [5, 6]. The comprehensive network, which is composed of various environmental factors affecting nursing students’ learning ability in the clinical learning process, is the core of the connotation of the clinical learning environment.

Several instruments have been developed to measure the clinical learning environment of nursing students, including the Clinical Learning Environment Scale (CLES) by Dunn S.V. in 1995 [7], the Student Evaluation of Clinical Education Environment inventory (SECEE) by Jecklin K.S. in 2000 [8], the Clinical Learning Environment Inventory (CLEI) by Dominic C. in 2001 [9], the Clinical Learning Environment and Supervision instrument (CLESI) by Saarikoski M. in 2002 [10], and the Undergraduate Clinical Education Environment Measure (UCEEM) by Strand P. in 2013 [11].

Briefly, the current measurement of the clinical learning environment mainly focuses on teaching security, emphasizing resource supply, ignoring the promotion of student development. Moreover, the International Workshop Agreement (ISO) launched the International Workshop Agreement “IWA 35:2020: Quality of Learning Environments for Students in Healthcare Professions—Requirements” in 2020, which proposed that the clinical learning environment be met in the context of frequent public health events for healthcare education providers in care settings [12]. However, the existing research has no consensus on measuring the clinical learning environment. Considering the comprehensive content of clinical learning, it is necessary to combine the development of medical education, pay attention to the core objectives of research, and clarify the specific scope and structure of the field. Moreover, the clinical learning environment is embedded in the social medical environment. The frequent occurrence of public health events has an enormous impact on the whole medical environment, and its effect on the clinical learning environment should also be considered. Therefore, based on the understanding of the clinical learning environment of nursing students in the current Chinese medical environment, this study developed the Clinical Learning Environment Scale for Chinese Nursing Students and tested its reliability and validity, providing an effective tool for evaluating the clinical learning environment of Chinese nursing students.

## Participants and methods

### Participants

#### Interviewees

This study used qualitative research methods to explore nursing students’ understanding of the nursing profession. The purposive sampling method was used, with sample selection based on the principle of maximum differentiation and information saturation. The inclusion criteria included full-time nursing undergraduates from universities in mainland China who had completed at least 6 months of clinical practice, had good expression ability, and provided informed consent and voluntary participation in the study. Ten nursing students, including three males and seven females from Jiangsu, Anhui, Henan, Shandong, and Ningxia provinces of China, were included in the study.

#### Panel of experts

Six experts were invited to provide expert consultation via in-depth interviews. The consultation aimed to revise and guide the structure, content and expression of the questionnaire items. The inclusion criteria for the experts were that they were familiar with the field, had a doctoral degree, held a senior title, and had undertaken relevant consulting work.

#### Participants in the quantitative study

Random sampling was used to select a total of 2,482 Chinese undergraduate nursing students. Of these, 293 prediction samples were used for project analysis and exploratory factor analysis, while the other 2,189 students were used as a validation sample for confirmatory factor analysis. The inclusion criterion for participants was full-time undergraduate nursing students from universities in mainland China. Additionally, they had to have completed at least six months of clinical practice and provided informed consent and voluntary participation in the study.

### Methods

#### Scale development

##### Theoretical basis

The Moos social environment theory was used as the framework for constructing the questionnaire. It is based on the study of the relationship between people and the environment from the perspective of social ecology and explains the adaptability of people to the environment from three dimensions: “personal development/goal orientation” “interpersonal relationship” and “system maintenance and system change” [13]. Johanna S. used the conceptual framework of the Moos social environment theory in 2012 to classify the items contained in the nine educational environment measurement scales, and the results supported that the framework could contain more than 90% of the environmental measurement elements [14]. This study uses the Moos social environment theory to elaborate on the adaptability between people and the environment. According to the division of environmental elements in the theoretical framework, the clinical learning environment is initially divided into three dimensions. “Goal oriented” refers to focusing on the teaching objectives and teaching plans of students’ clinical learning stage based on the perspective of personal development. “Interpersonal relationship” refers to the experience brought about by the interaction between learners and others and the emotional support gained therefrom. “Learning support” refers to the physical factors, cultural factors and innovation that support the development of students’ clinical learning.

##### Literature review

The CLES, SECEE, UCEEM, CLESI, CLEI, and IWA 35:2020 were used in combination with the current clinical situation in China to preliminarily construct the content framework for the scale.

##### Qualitative interviews

A semistructured interview was conducted with 10 Chinese nursing students in October 2022 to determine their understanding of the clinical learning environment. The interview topics included the following: (1) Please describe your clinical learning environment. (2) What are you satisfied with your clinical learning environment? (3) What are you not satisfied with your clinical learning environment? The interview data were coded and classified by using the three-level coding procedures of open coding, main axis coding and selective coding. According to the coding results and referring to the items of the existing scale, an item pool of the scale content was formed, and the initial scale containing 3 dimensions and 23 items was compiled.

##### Expert consultation

Expert consultation was conducted in the form of interviews in November and December 2022 to review the content validity of the questionnaire. The experts were asked to evaluate whether the variables of the questionnaire fully reflected the problems of this study, whether the dimension division of each variable was reasonable, whether the expression of each item was in line with practice, and whether the expression of each item accurately expressed the intended meaning. Seventeen items were unanimously agreed upon by six experts, four items were revised by one expert, one item was deleted by three experts, and one item was deleted by four experts. After receiving expert suggestions, two items were deleted, and four items were revised according to their opinions.

##### Investigation and verification of the predictive version

In December 2022, the predictive version of the Clinical Learning Environment Scale for Chinese Nursing Students was used to survey 293 nursing students, and 255 valid questionnaires were collected, for an effective response rate of 87.0%. The criteria for excluding invalid questionnaires are outlined in section “Quality management”. At this stage, the items of the scale were screened, and exploratory factor analysis was conducted to form the final version of the clinical learning environment scale for Chinese nursing students.

##### Investigation and analysis of the validated version of the scale

From January to March 2023, the final version of the Clinical Learning Environment Scale was used for Chinese nursing students to survey 2189 nursing students, and 1582 valid questionnaires were collected, for an effective response rate of 72.3%. The criteria for excluding invalid questionnaires are outlined in section “Quality management”. Confirmatory factor analysis was used to verify the structural validity of the scale.

#### Item analysis

Three methods were employed for item analysis [15]: ① Critical ratio: The independent sample T test of SPSS 22.0 software was used to calculate the critical ratio > 3, and *P* < 0.05 was used as the cutoff value for each item according to the 27% grouping method. ② Correlation coefficient: the correlation coefficient between each item score and the total score was > 0.4, and *P* < 0.05. ③ Cronbach’s α coefficient: The correlation between the revised items and the total score was greater than 0.40, and the total Cronbach’s α coefficient of the scale did not increase after deleting the items. Any items that did not meet the above criteria were deleted.

#### Exploratory factor analysis

When the Kaiser–Meyer–Olkin (KMO) value exceeded 0.8 and Bartlett’s sphericity test P was less than 0.05, it was determined that there was a good relationship between variables, which indicates that the predictive scale was suitable for exploratory factor analysis. The principal component analysis method was adopted, and the maximal variance rotation was selected for rotation. The item selection criterion was a factor loading of not less than 0.50, and the factor extraction criterion was a factor eigenvalue greater than 1 and a number of items larger than 3 [16].

#### Confirmatory factor analysis

A structural equation model was used for verification, and the verification indices included the absolute fitness index, incremental fit index, and parsimony fit index. When the GFI, AGFI, NFI, IFI, and CFI values were greater than 0.90, the PCFI and PNFI were greater than 0.5, the RMSEA was less than 0.1, and the RMR was less than 0.05, this indicated a reasonable model fit. The composite reliability (CR) and average variance extracted (AVE) were used to test the convergent validity of the questionnaire, where CR > 0.7 and AVE > 0.5 indicated good convergent validity for each factor of the variable and higher intrinsic quality of the model [17].

### Quality management

In qualitative research, the interviewers received qualitative research courses during their doctoral studies and were proficient in relevant methods. In the prediction and verification stages of scale development, the purpose of the study was to introduce the participants, and an informed consent process was carried out. To ensure the objectivity and authenticity of the data, the survey excluded questionnaires that took less than two minutes or more than ten minutes to answer, had all consistent responses, or were not answered as directed. While filling out the questionnaire, explaining to students the purpose of the study, emphasizing that the purpose of the questionnaire is to collect genuine feedback rather than assess individual performance, and ensuring that it will not have adverse effects on them to guarantee the objectivity of students’ responses.

### Statistical analysis

SPSS 22.0 software was used to analyze the data. Pearson correlation analysis was used to calculate the correlation between each item and the scale, and Cronbach’s α was used to test for homogeneity. Factor analysis was used to explore the internal structure of the scale, and principal component analysis combined with the varimax rotation method was used to extract common factors. The AMOS 26.0 structural equation model was used for confirmatory factor analysis with a significance level of a = 0.05 (two-tailed).

## Results

### Basic information on the prediction samples and verification samples

The characteristics of the participants in the prediction and verification versions of the survey are shown in Table 1.

**Table 1:** Characteristics of the prediction and verification samples

Characteristics		Prediction sample	Verify the sample
Gender	Male	21	240
	Female	234	1342
Source	City	89	469
	Urban or Rural areas	166	1113
School type	Double First- Class Universities	52	333
	Other Universities	203	1249
Academic performance in school	Top 30%	98	598
	Ranked 30–60%	132	763
	After ranking 60%	25	221
Whether to choose nursing specialty independently	Yes	138	978
	No	117	604

### Item analysis results

① The CR value of each item ranged from 9.007 to 20.180, and the *p* value was less than 0.05. ② The correlation coefficient between the score of each item and the total score of the scale ranged from 0.602 to 0.896, and the *P* value was less than 0.05. ③ The correlation coefficient between the revised items of each item and the total score was between 0.570 and 0.882. Therefore, based on the results of the item analysis, all 21 items were retained.

### Prediction sample analyses

The KMO value of the scale was 0.962, and the chi-square value of Bartlett’s sphericity test was 4908.278 (*p* < 0.001), indicating that the scale was suitable for exploratory factor analysis. The principal component analysis method was used to extract three factors with eigenvalues greater than 1. 0, and the cumulative explained variance was 71. 500%. According to the item deletion principle of exploratory factor analysis, item 6 and item 10 have dual factor loads at the same time and should be deleted. Finally, 19 items were reserved for the scale, including 5 items related to “goal orientation”, 4 items related to “interpersonal relationships” and 10 items related to “learning support” (Table 2). The total Cronbach’s α of the scale was 0.964, higher than 0.9, and the Cronbach’s α of each dimension was 0.873, 0.859 and 0.954, higher than 0.8.

**Table 2:** Exploratory factor analysis of the “Chinese Nursing Students’ Professional Self-concept Scale”

Item	Factor load		
Items 1: I am able to perform sufficient clinical rotations to achieve learning objectives			0.527
Items2: I have a specific job assignment			0.574
Items3: My work assignments are appropriately challenging for my level of knowledge			0.783
Items4: I am able to experience care based on contemporary evidence-based practice concepts			0.726
Items5: I can apply the knowledge learned in school to clinical practice.			0.719
Items 7: The teacher’s expectations of my performance are realistic.		0.678	
Items 8: With the increase of skills, the teacher gave me more opportunities to operate at bedside.		0.730	
Items 9: I consider myself a member of the internship department, and I have a good relationship with everyone.		0.586	
Items 11: Patients are willing to accept my care and give frequent feedback		0.787	
Items 12: The teacher provided me with enough guidance to acquire new knowledge.	0.581		
Items 13: Medical ethics training runs through the whole process of clinical learning	0.549		
Items 14: The hospital has a perfect nursing teaching management system	0.635		
Items15: There are a lot of meaningful learning situations in the ward.	0.677		
Items 16: Clinical teaching methods are diverse and innovative	0.725		
Items 17: I can negotiate my own schedule	0.584		
Items18: The hospital has good communication channels to listen to students’ opinions and make corresponding improvements.	0.819		
Items 19: My psychological stress at work can be noticed and supported effectively	0.771		
Items20: The hospital can determine the roles and responsibilities of the students and does not rely on the students to carry out the core work content.	0.826		
Items 21: The hospital can effectively deal with all kinds of emergencies that threaten the safety of students	0.766		
Eigenvalue	5.986	3.833	3.766
Explained Variance%	31.506	20.172	19.822
Cumulative explained variance%	31.506	51.678	71.500

### Analyses of the validation sample

**Fig. 1:**
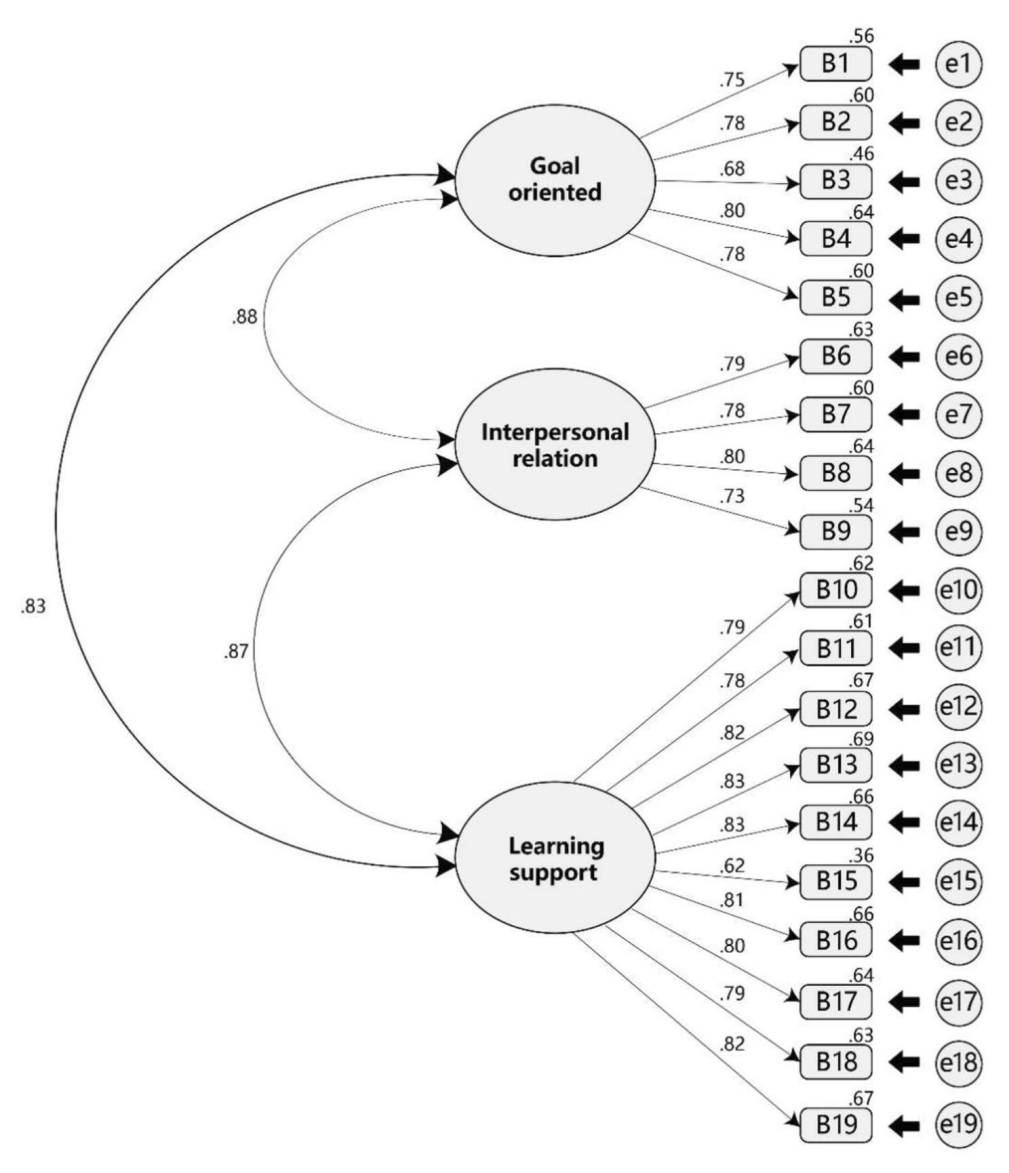
Confirmatory factor analysis model of the “Clinical Learning Environment Scale” for Chinese nursing students

The factor loading of 19 items ranged from 0.62 to 0.83, which was higher than the basic requirement of 0.5 (Fig. 1). Regarding the model fitting indicators, the GFI was 0.848, the AGFI was 0.806, the RMSEA was 0.090, the RMR was 0.041, the NFI was 0.910, the IFI was 0.916, the CFI was 0.916, the PCFI was 0.798, and the PNFI was 0.793. Except for the GFI and AGFI, which failed to reach 0.9, all the other indicators met the adaptation criteria; however, according to the views of Segars and other scholars [18], the GFI and AGFI values are acceptable if they are greater than 0.8. Therefore, no corrections to the model were needed.

The convergent validity of the questionnaire was tested using composite reliability (CR) and average variance extraction (AVE). The composite reliabilities of the three dimensions were 0.870, 0.858, and 0.943, which were all greater than 0.7, and the average variance extractions were 0.574, 0.603, and 0.625, which were all greater than 0.5. The absolute values of standardized factor loadings of each item ranged from 0.617 to 0.831, all of which were greater than 0.6 and significant. (Table 3).

**Table 3:** Summary of model parameter estimation of the “Clinical Learning Environment Scale”

Latent variable	Items	Parameter significance estimation				Std.	SMC	CR	AVE
		Unstd.	S.E.	Z value	*P*				
Goal oriented	Items 1	1.000			***	0.749	0.560	0.870	0.574
	Items 2	0.996	0.032	31.019	***	0.778	0.605		
	Items 3	0.940	0.035	26.739	***	0.678	0.460		
	Items 4	1.123	0.035	32.036	***	0.801	0.642		
	Items 5	0.932	0.030	30.913	***	0.775	0.601		
Interpersonal relation	Items 6	1.000			***	0.792	0.627	0.858	0.603
	Items 7	1.102	0.033	33.247	***	0.777	0.604		
	Items 8	1.195	0.035	34.497	***	0.800	0.641		
	Items 9	0.948	0.031	31.025	***	0.735	0.540		
Learning support	Items 10	1.000			***	0.788	0.621	0.943	0.625
	Items 11	0.999	0.029	34.439	***	0.779	0.607		
	Items 12	1.066	0.029	36.789	***	0.819	0.671		
	Items 13	1.113	0.030	37.514	***	0.831	0.691		
	Items 14	1.130	0.030	37.246	***	0.827	0.683		
	Items 15	0.920	0.036	25.873	***	0.617	0.380		
	Items 16	1.244	0.034	36.438	***	0.813	0.661		
	Items 17	1.205	0.034	35.647	***	0.800	0.640		
	Items 18	1.182	0.034	35.262	***	0.793	0.629		
	Items19	1.131	0.031	36.623	***	0.816	0.666		

* * *:*P* < 0.01

## Discussion

The clinical learning environment is a synthesis of various factors that affect the learning of nursing students in the process of clinical learning, and it is an interactive network composed of various interrelated and interacting factors. Nursing is essentially a practical discipline, and clinical learning is a key step in the training of nursing students. Therefore, the clinical learning environment has an important impact on the professional development of nursing students and must be closely monitored.

Based on the literature, the measurement of the clinical learning environment in this study is based on the concept of process quality, focusing on the experience perception and evaluation of nursing undergraduates in the process of the clinical learning environment to reflect the current situation of students’ clinical learning environment and its potential impact on students. Therefore, this research focuses on the interaction process between people and the environment, and the Moos social environment theory divides the clinical learning environment into three structural dimensions: “goal-oriented” “interpersonal relationship” and “learning support” The researcher drew on existing scales such as the CLES, SECEE, UCEEM, CLESI, and CLEI, referred to the IWA 35:2020 document issued by the ISO, and combined them with some qualitative interviews to compile the first draft of the Clinical Learning Environment Scale for Chinese Nursing Students.

Subsequently, 255 and 1582 Chinese nursing students were selected as prediction and verification samples, respectively, and the final version of the Clinical Learning Environment Scale for Chinese Nursing Students, containing three dimensions and 19 items, was finalized through item analysis, exploratory factor analysis, and confirmatory factor analysis. The results of reliability and validity testing showed that the Cronbach’s α coefficient of the scale was 0.964, which was significantly greater than 0.7. Furthermore, the standardized factor loads of all the measurement items were greater than 0.6, indicating that each item of the scale had a good measurement relationship, and the AVE of each dimension was greater than 0.50, indicating that the convergent validity of each factor of the scale was good. Additionally, the fitness indices, such as GFI, NFI, IFI, CFI, AGFI, PCFI, PNFI, RMSEA, and RMR, all met the standard, indicating that the model fit well.

Clinical learning is the core link of nursing personnel training and has generally been valued by scholars; however, existing research has focused more on teaching security and the supply of resources, ignoring the promotion of student development. Moreover, the frequent occurrence of public health events has also led to higher requirements for the clinical learning environment of nursing students, while scholars have not paid enough attention to this topic. In addition, indigenous Chinese medical culture affects students’ understanding of the clinical learning environment. According to the training objectives of nursing students in China combined with the actual medical environment, this study not only supplemented the development of students, security and ideological guidance but also emphasized teaching resources to form a clinical learning environment composed of three subdimensions—goal oriented, interpersonal relation and learning support—which is more in line with the reality of the current local clinical learning environment in China.

During the process of completing this questionnaire, students may be involved in factors related to their future career development, awareness of the importance of learning support, and emotional venting regarding the clinical learning environment. All these factors could potentially influence their responses. Therefore, in order to enhance the consequential evidence of this study, we specifically emphasized the purpose and neutrality of this research during implementation, ensuring that it will not have adverse effects on the participants.

In summary, the reliability and validity of the Clinical Learning Environment Scale for Chinese Nursing Students compiled in this study meet the relevant requirements and can be applied to the measurement of the professional self-concept of Chinese nursing students.

## Limitations

However, this study has several limitations. Firstly, Because the sample representativeness of the test is not wide enough, it may have a certain impact on the comprehensiveness of the research conclusions. Future research can expand the coverage of the survey and further test the implementation of the scale. Secondly, By considering the workload to fulfill the questionnaire and the response rate, outside scale was not included in the survey, resulting in the lack of concurrent validity of the Clinical Learning Environment Scale for Chinese Nursing Students. Thirdly, although the survey was anonymity, this is a self-reported scale, social desirability bias might exist.

## Conclusion

Based on Moos social environment theory, this study developed the Clinical Learning Environment Scale for Chinese Nursing Students with reference to the literature and the content of qualitative interviews. The reliability and validity of the scale were tested by an empirical study, which provided an effective tool for measuring the clinical learning environment of Chinese nursing students.

## Data Availability

The datasets used and analyzed during the current study are available from the corresponding author upon reasonable request.
